# Silver ion bioreduction in nanoparticles using *Artemisia annua* L. extract: characterization and application as antibacterial agents

**DOI:** 10.1186/s13568-020-01002-w

**Published:** 2020-04-07

**Authors:** Anush Aghajanyan, Lilit Gabrielyan, Robin Schubert, Armen Trchounian

**Affiliations:** 1 Department of Biochemistry, Microbiology and Biotechnology, Biology Faculty, Yerevan State University, 1 A. Manoukian Str., 0025 Yerevan, Armenia; 2 Department of Medical Biochemistry and Biotechnology, Russian-Armenian University, 123 H. Emin Str., 0051 Yerevan, Armenia; 3 European X-Ray Free-Electron Laser Facility GmbH, Holzkoppel 4, 22869 Schenefeld, Germany

**Keywords:** Green synthesis, Plant extract, Silver nanoparticles, Antibacterial activity, Bacterial cell growth

## Abstract

The biological synthesis of metal nanoparticles using plant extracts with defined size and morphology is a simple, nontoxic and environmentally friendly method. The present study focused on the synthesis of silver nanoparticles (Ag NPs) by *Artemisia annua* L. extract as reducing and stabilising agent. The Ag NPs function, as antibacterial agents, is with that they are further used in human therapy. The effects of pH and temperature on the synthesis of NPs were characterized by UV-absorption spectroscopy and shown by surface plasmon resonance (SPR) band at 410 nm. NPs’ size and morphology were measured by transmission electron microscopy (TEM) and dynamic light scattering (DLS). TEM images showed that Ag NPs were in a nano-sized range (20–90 nm) and had spherical shape. Our findings demonstrated that lower concentration (100 µg mL^−1^) of the biogenic Ag NPs exhibited antibacterial activity against Gram-negative *Escherichia coli* BW 25113 and Gram-positive *Enterococcus hirae* ATCC 9790.

## Introduction

**Fig. 1:**
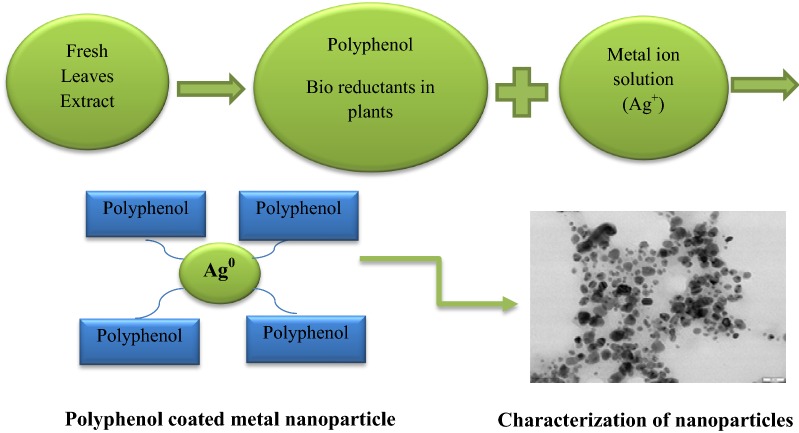
Pattern of green synthesis. The chemical reaction of NPs synthesis includes several steps. Polyphenols convert positive Ag^+^ into the zero Ag^0^ valent metal, and in the last step of green synthesis the polyphenols coat metal NPs and affect the morphology and size of NPs

Recently, with the development of modern technologies of the nanomaterial synthesis, there was interest in studying the properties of metals at ultra-disperse range as a powder, solution and suspension. As a rule, the nanoparticles (NPs) may easily form complexes with different substances due to their high chemical activity. These complexes have new properties such as good solubility and high biological activity. In this regard, the water dispersion of metal NPs that was obtained by biochemical synthesis using plants shows the ability to absorb, accumulate and restore inorganic metal ions from the environment (Rastogi et al. 2017; Griffin et al. 2018). The various organic components, particularly, secondary metabolites that are present in plant tissues are able to act as stabilizing and reducing agents in the process of NPs synthesis (Kulkarni and Muddapur 2014; Asif et al. 2015; Chokkareddy and Redhi 2018; Rahman et al. 2019). Reduction and formation of NPs occur in the water core of micelles formed by surfactant molecules using natural biologically active substances such as plant pigments from the flavonoid group which ensures long-term stability of NPs and makes this process as safe as possible for the environment. The highest activity, and final morphology of NPs is ultimately reached in the last step of green NPs synthesis, when they are coated with plant metabolites (polyphenols, tannins, terpenoids etc.). Many biological systems of plants can convert inorganic metal ions into metal NPs through the reductive abilities of secondary metabolites present in these organisms. The ability of plants to accumulate and detoxify heavy metals is well proved (Song and Kim 2009; Chokkareddy and Redhi 2018). Bioactive compounds of plants such as polyphenols, flavonoids, vitamin C, alkaloids and terpenoids, reduce silver (Ag) salts from positive oxidation state (Ag^+^) to zero oxidation state (Ag^0^); the mechanism for reduction of Ag^+^ to Ag^0^ is shown (Fig. 1). Secondary metabolites present in the plant extract affect the size and shape of metallic NPs. These biologically active compounds possess antioxidant activity and are of great interest in the biomedical field as alternative antibacterial agents (Gouveia and Castilho 2013; Tarahovsky et al. 2014; Asif et al. 2015).

In recent years, metallic NPs, particularly Ag are used in medicine as antimicrobial (Nadeem et al. 2017; Rao and Tang 2017; Qing et al. 2018; Roy et al. 2019) and anti-tumor agent (Ovais et al. 2016; Sahu et al. 2016, Barabadi et al. 2019). Ag has much more pronounced antimicrobial effect compared to the other antibiotics. It has a similar effect on antibiotic-resistant strains of bacteria and has been used in medical sphere (Silva et al. 2010; Seil and Webster 2012). The microbicidal effect of Ag is unique, because it is directed to damage the bacterial cell structures. This indicates the perspective for using this metal as an antibacterial agent (Seil and Webster 2012). However, the inorganic silver (Ag^+^) can be toxic for the living cells, so when exposed to Ag^+^ the plant uses an oxidizing and reductive pathway of detoxification, and as a result, elementary particles are formed (Rahman et al. 2019). The coating of Ag NPs with secondary metabolites reduces toxicity (Kulkarni and Muddapur 2014; Sahu et al. 2016; Rahman et al. 2019). The interaction of NPs with plants causes some morphological and physiological changes depending on the properties of NPs. In contrast to chemically synthesized NPs, the green synthesis has a minimal impact on the environment; moreover, chemically synthesized NPs are expensive and dangerous for the environment and living organisms (Chokkareddy and Redhi 2018). Therefore, there is an urgent need for an alternative, cost-effective and environmentally friendly method for NPs production.

Recently, Ag NPs have attracted researchers’ interest due to unique catalytic properties, moreover, among the noble metals the Ag NPs are the most studied due to their antibacterial efficacy (Kelly et al. 2003; Kulkarni and Muddapur 2014; Nadeem et al. 2017; Rao and Tang 2017; Roy et al. 2019). These NPs are used as an alternative antibiotic therapy, and their therapeutic effects depend on important aspects, such as particle size, shape and concentration. The size of NPs is one of the most important properties for antibacterial activity. Particular small NPs have high antibacterial activity (Waclawek et al. 2018; Vega-Jimenez et al. 2019) due to their large surface area and high intracellular penetration (Roy et al. 2019). Preferably, the size of NPs must be lower than 50 nm to acquire effective antibacterial activity, however very small NPs (< 10 nm) are toxic due to their higher intracellular bioavailability. The mechanism showed that the attachment of NPs on cell membrane led to membrane damage, as a result of increased membrane permeability, which eventually led to cell death (Gabrielyan and Trchounian 2019; Roy et al. 2019).

The herbs are not only interesting as natural sources for human consumption but they are also easily available, non-toxic and they are important resources due to their complex molecular composition. Indeed, the plant extracts are rich with bioactive compounds which have an important role as reducing agents in NPs synthesis (Ovais et al. 2016; Griffin et al. 2018; Roy et al. 2019). Various parts of plants, such as leaves, roots, seeds and stems are widely used for the synthesis of metal NPs; nevertheless, the leaves are the most commonly used parts of plants. Due to the growing interest of application of medicinal plants, it is important to study therapeutic effects of widely used medicinal plants. As a consequence of green NPs synthesis, the NPs reach the highest possible activity when they are covered by plant metabolites. The biological approach of NPs synthesis using plants is a comparatively fast, cheap, simple, energy-efficient and eco-friendly method (Griffin et al. 2018). Moreover, particles obtained from plant extracts have good quality, small size and spherical shapes (Kelly et al. 2003; Waclawek et al. 2018; Vega-Jimenez et al. 2019). On the other hand, there is a concern about possible toxic effects of Ag in humans; hence our findings are directed towards the search for new antibacterial nanomaterial with non- toxic properties for human.

Among the medicinal plants, *Artemisia annua* L. has been identified as traditional herbal remedy against inflammatory disease, infections by bacteria and viruses (Ram 2011; Li et al. 2015; Waclawek et al. 2018). However, over the past decade, the *A. annua* has been used in the treatment not only against viruses, but also as an anti-diabetic, anti-tumor agent (Schramek et al. 2010; Li et al. 2015). *A. annua* is a plant used for many centuries in Armenian folk medicine for the treatment of different diseases. It has been reported that the most important bioactive metabolite of *Artemisia* is the sesquiterpene lactone artemisinin (Schramek et al. 2010; Ram 2011). The leaves have a high content of essential oil which has antifungal and antimicrobial activities (Gouveia and Castilho 2013). Therapeutic effect and properties of *A. annua* can differ according to the geographical location and how the plant is grown. Interest in the potential applications of this plant is still increasing and in the present study we investigated *A. annua* plant which has been grown in hydroponics conditions. The antioxidant capacity of this plant is associated with the flavonoid content and diverse bioactive compounds which can act as both reducing and stabilizing agents in NPs synthesis process (Raveendran et al. 2003; Schramek et al. 2010; Li et al. 2015). Nevertheless, the mechanism of NPs formation by green synthesis method is still not understood. Stability and toxicity of Ag NPs also should be investigated.

The present study aimed to investigate the properties and biological activity of “green” synthesized Ag NPs obtained from *A. annua.* For reveal the antibacterial activity of these NPs, the growth properties and susceptibility of Gram-negative *Escherichia coli* and Gram-positive *Enterococcus hirae* in the presence of these Ag NPs have been determined.

## Materials and methods

### Plant material, growth condition and preparation of plant extract

*Artemisia annua* L. was grown using hydroponics method; dry material was supplied by the Institute of Hydroponic Problems, National Academy of Sciences, Yerevan (Armenia). Sprouts of this plant were transplanted in conditions of classical hydroponics (seating density was 1 plant per m^2^). Particles of volcanic slag with diameter of 3–15 mm served as substrate for plant, nutrition solution was used as described (Davtyan 1980).

5 g of *A. annua* leaf powder was added to 100 mL of triple distilled water in Erlenmeyer flask and mixture was shaken at 60 °C, 150 rpm during 2 h. The solution was filtered through Watman filter paper and extract was used for metal NPs synthesis.

### Biosynthesis of Ag NPs

For the synthesis of Ag NPs 5 mL of aqueous extract of *A. annua* was added to 45 mL of 1 mM AgNO_3_. The mixture has been shaken at room temperature for 50-60 min. The study was conducted at a temperature 21 °C (room temperature) and at pH 7.0. The effects of various temperature (40 °C, 60 °C) and pH (3.0, 5.0, 7.0 and 9.0) were studied. To test the influence of the pH value from 3.0 to 9.0 small amount of 0.1 N HCl and 0.1 N NaOH was added to the reaction mixture. The color change to dark brown in the reaction mixture indicated the formation of Ag NPs were left to dry covered overnight. The synthesized Ag NPs were used for the assessment of antibacterial efficacy.

### Characterization of Ag NPs

The UV–visible absorption of Ag NPs suspension was used to confirm the formation of nanoparticles. 2 mL of Ag NPs suspension was analyzed using spectrophotometer (Genesys 10S UV–VIS-Thermo Fisher Scientific and UV 2700 Shimadzu). The absorption of the sample was recorded in the wavelengths ranging from 200 to 800 nm, at a resolution of 1 nm, with 1 cm path length quartz cuvettes to obtain plasmonic curves. Transmission electron microscopy (TEM, 200 keV, JEM 2100-Plus Jeol) analysis was performed on applied samples to investigate the formation of Ag NPs and their shape (operated at 200 keV accelerating voltage) with selected area electron diffraction (SAED, 250 mm camera length). For recording the TEM image 4 µL sample was applied on freshly glow-discharged carbon-coated cooper grid, incubated for 2 min, blotted and dried. The hydrodynamic diameter of synthesized NPs (1:10,000 diluted in water) was measured by Nanosight (NS300 Malvern Panalytical) by injecting 200 µL of sample into the flow cell. Three measurement movies were recorded and the sample was advanced in the flow cell between each measurement to measure different sample subsets. The hydrodynamic radius of particles (1:100 diluted in water) was measured using dynamic light scattering (DLS, Spectrolight 600 XtalConcepts) by applying 1 µL of sample into a 96 well plate, topped with paraffin oil to avoid evaporation over time.

### Evaluation of antibacterial activity of Ag NPs

Gram-negative and Gram-positive bacteria were used for studying the antibacterial potential of the biogenic Ag NPs. The antibacterial activity of Ag NPs was determined against two wild type strains (*Escherichia coli* BW 25113 and *Enterococcus hirae* ATCC 9790). *E. coli* was received from Keio collection (Tsuruoka City, Yamagata, Japan), and *E. hirae* was supplied by Prof. M. Solioz (Department of Clinical Pharmacology, University of Bern, Switzerland). The bacteria were cultivated in 150 mL flasks at 37 °C with peptone and tryptone growth media, respectively (Poladyan et al. 2013). Strictly anaerobic conditions were maintained throughout the experiment. The growth of bacteria was determined by optical density (OD) which was measured at 600 nm using spectrophotometer. Ag NPs with concentration from 100 to 450 mg mL^−1^ were added into the bacterial growth medium.

### Assay of bacterial susceptibility to Ag NPs

To study the susceptibility of *E. coli* and *E hirae* to NPs bacteria were cultivated in the presence of the investigated samples (100 µg mL^−1^). Various dilutions of bacterial suspension (10^6^–10^8^) were applied (Gabrielyan et al. 2019). 100 µL of each sample was spread on nutrient agar plates. Finally, the growth of bacterial colonies was estimated after 24 h incubation at 37 °C. The number of bacterial viable colonies in each sample were counted for determination of the number of colony forming units (CFUs). Nutrient agar plates contained appropriate peptone and tryptone media with 1.5% bacteriological agar; experiments were conducted in anaerobic conditions. The number of bacteria was calculated by T = 10 × n × 10 ^m^, where *n* is the number of viable bacterial colonies, *m*—number of dilutions (Gabrielyan et al. 2019).

Tryptone, glucose, DCCD were obtained from Sigma Aldrich (USA); peptone, Tris (aminomethane) from Carl Roth GmbH (Germany). Triple distilled water was used throughout the experiment. Other chemicals of analytical grade were used.

### Statistical analysis

Each experiment was performed thrice, and data are mean ± SE (n = 3). Student’s *t*-test was selected for statistical analysis and evaluation of statistically significant differences of experimental data between different series. p ≤ 0.05 for difference between the mean values was considered as statistically significant.

## Results

### Characterization of Ag NPs by UV/Vis spectrophotometer

**Fig. 2:**
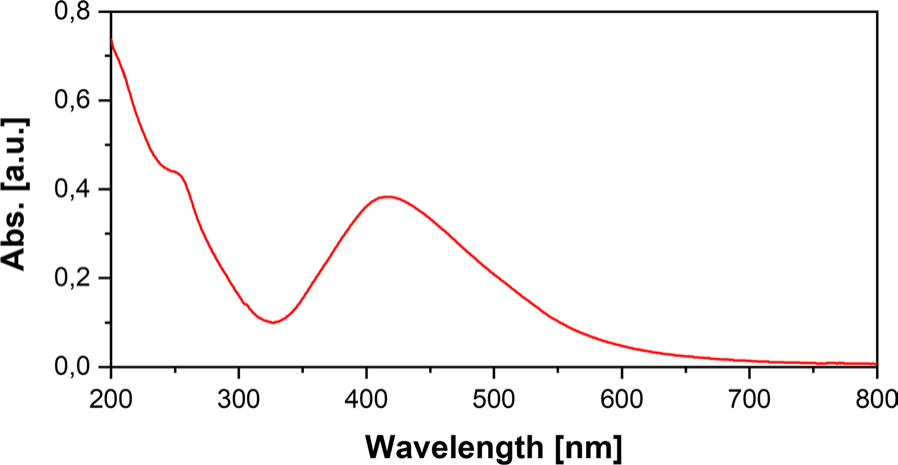
UV-visible spectrum of green synthesized Ag NPs. 5% extract of *A. annua* and 1 mM AgNO_3_ at 21 °C and pH 7.0

The optical absorption of Ag NPs is a common method for determination of formation and stability of NPs. The formation of Ag NPs in the reaction mixture was verified by UV–VIS absorbance method that shows the surface plasmon resonance band (SPR). The result of UV/Vis spectrum indicated the SPR peak at 410 nm which is a characteristic peak of Ag NPs (Fig. 2). This peak is a characteristic peak for Ag NPs (Iravani and Zolfaghari 2013; Kulkarni and Muddapur 2014; Ansari et al. 2018).

### Effect of pH

**Fig. 3:**
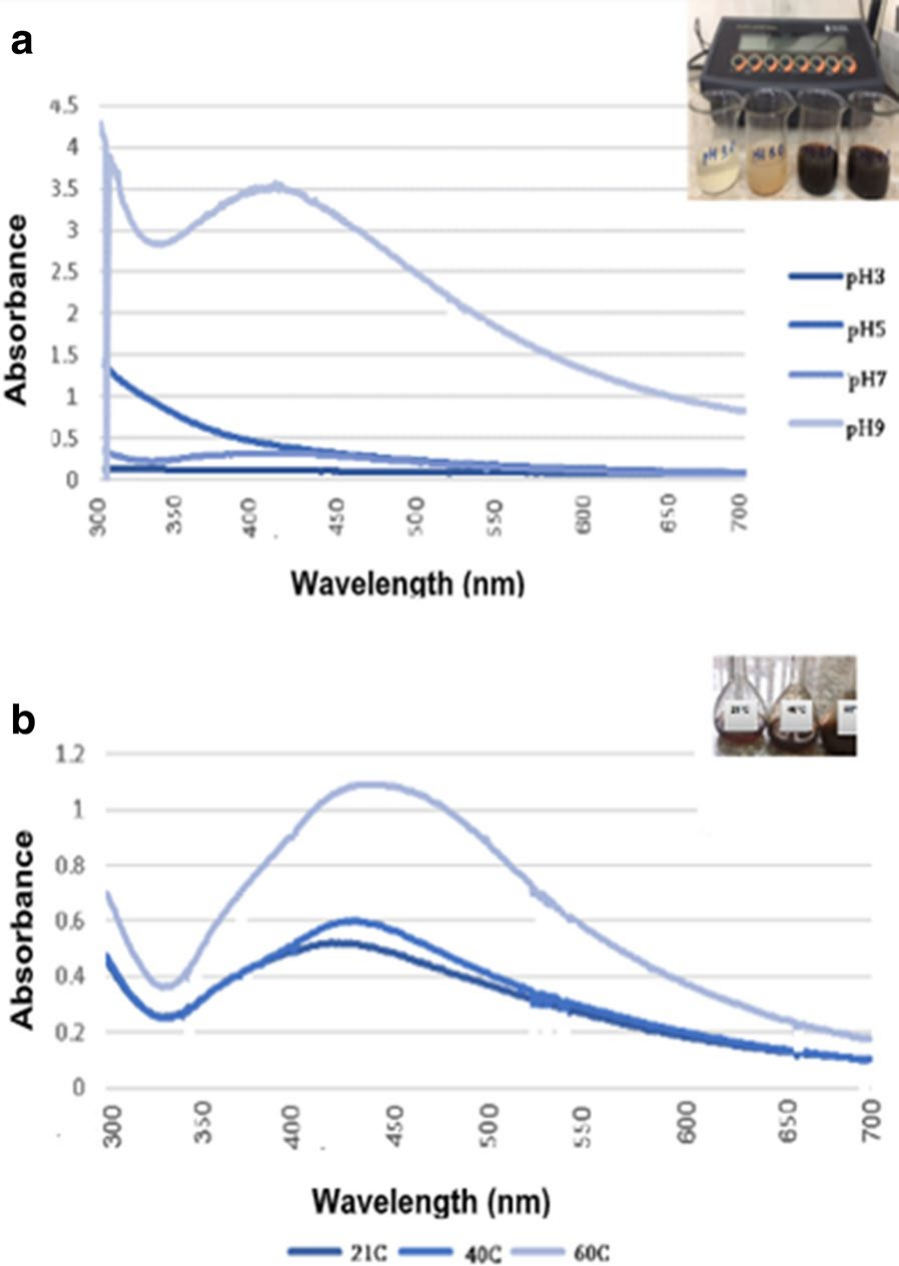
UV-visible spectrum of green synthesized Ag NPs with different initial pH values (**a**) and at different temperature (**b**). For the others, see the legends to Fig. 2

Formation, size and shape of NPs depend on important biochemical factors such as pH, temperature and concentration. pH is a crucial physicochemical factor that affects the biogenic synthesis, size and morphology of NPs (Njagi et al. 2011; Prasad 2014; Singh and Srivastava 2015; Polyakova et al. 2017; Waclawek et al. 2018). The pH modification leads to charge conversion in the plant metabolites, provokes the resolving of metal ions, thereby altering the size and morphology of synthesized NPs (Polyakova et al. 2017). The reduction of Ag^+^ to Ag^0^ was performed at pH 3.0, 5.0, 7.0 and 9.0 with *A. annua* extract. Our data showed that formation of NPs occurs at pH 7.0 and 9.0. The absorbance of the solution was measured by the spectroscopic approach (Fig. 3a). No Ag NPs were formed at pH 3.0 and 5.0. The reaction mixture turned dark brown when Ag^+^ was reduced at neutral and alkaline pH (7.0 and 9.0), thus the small size of NPs formed. Iravani and Zolfaghari (2013) had similar results and reported that pH plays an important role for the synthesis of NPs and the size and shape of NPs depend on pH value. Several researches reported that at low pH value instead of nucleation of NPs their aggregation was observed (Iravani and Zolfaghari 2013; Polyakova et al. 2017; Ansari et al. 2018). Thus, the neutral and alkaline pH value promotes the formation of NPs with smaller diameter.

### Effect of temperature

The temperature also is one of the crucial factors affecting nucleation and size of NPs (Njagi et al. 2011; Waclawek et al. 2018). The reaction mixture was heated to different temperatures (40 °C and 60 °C) using a water bath and was kept at room temperature (21 °C). The obtained spectra of the samples showed that the formation of NPs occurs for a short time at higher temperatures (40 °C and 60 °C) (Fig. 3b). Perhaps, it can be due to the increase of the formation of colloidal Ag NPs. The formation of NPs also occurs at room temperature, however their formation is in much slower rate and the surface plasmon resonance band is not sharp compared to the high temperatures which showed a faster rate of synthesis (Fig. 3b).

### TEM study

**Fig. 4:**
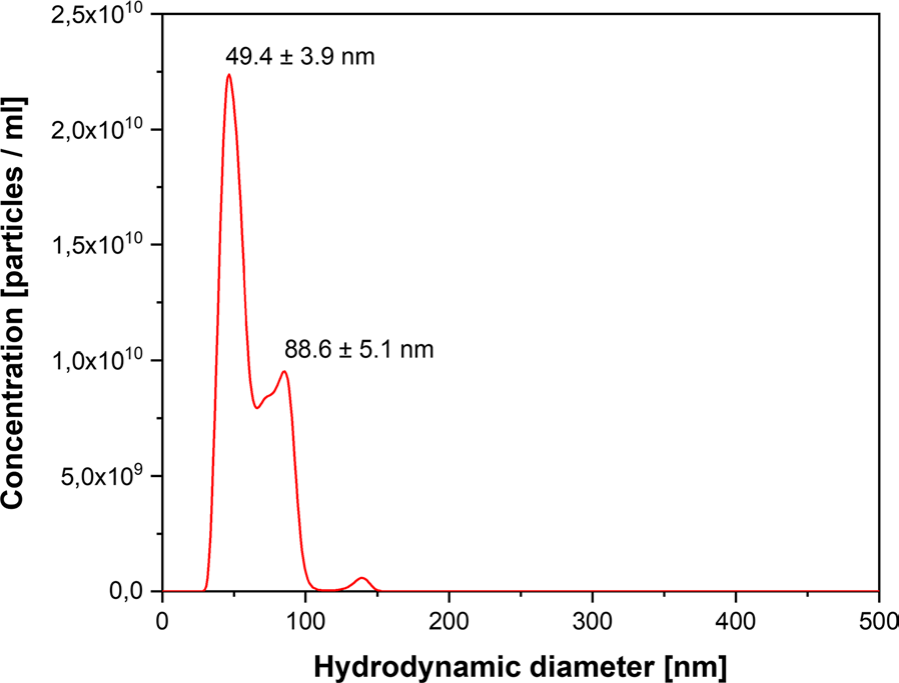
Hydrodynamic diameter of green synthesized Ag NPs by Nanosight analysis. Integrated Ag NP concentration of 7 × 10^11^ particles mL^−1^ (1:10,000 diluted in water) was revealed. For the others, see the legends to Fig. 2

**Fig. 5:**
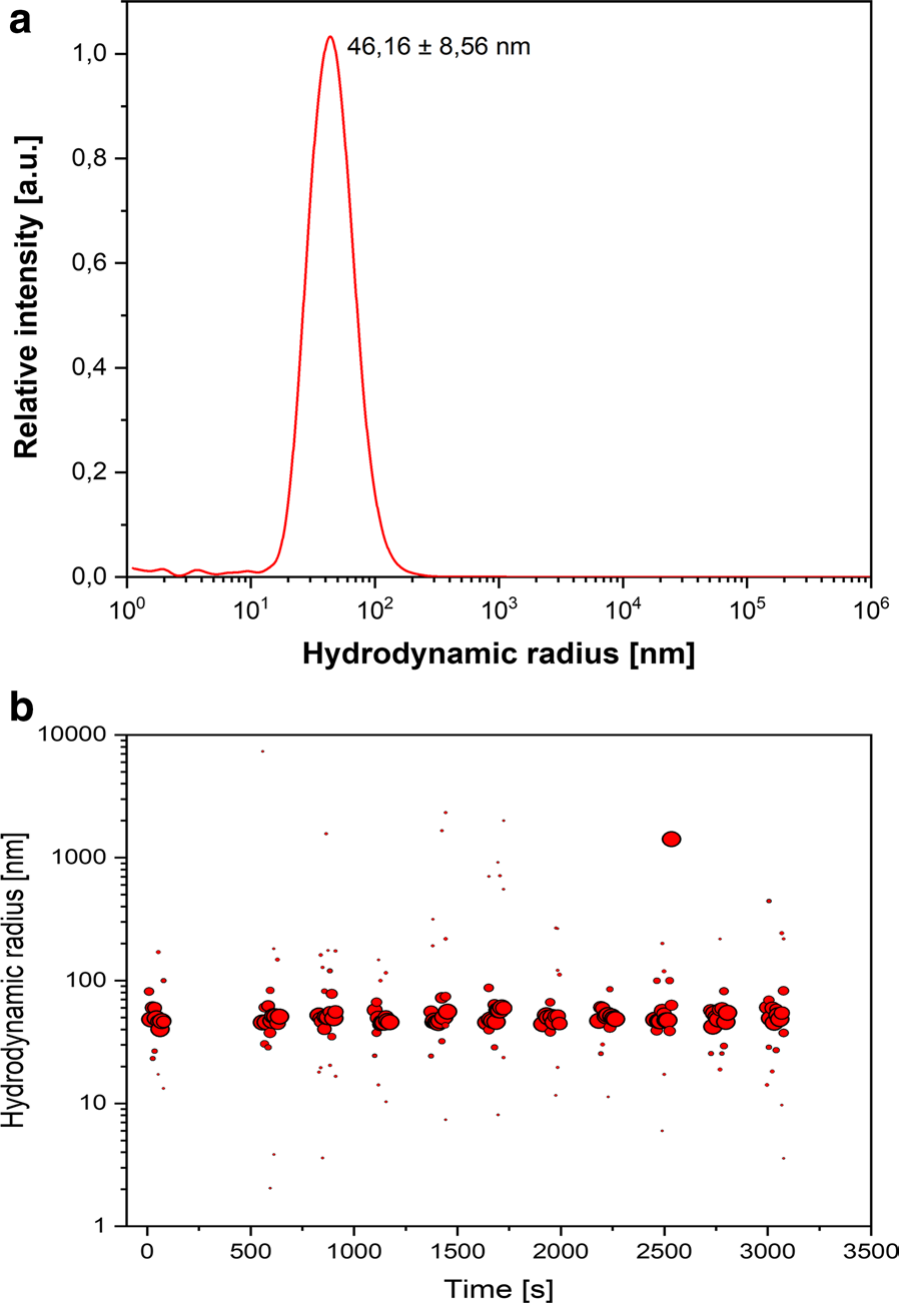
Dynamic Light Scattering (DLS) measurement of biosynthesized Ag NPs. For the others, see the legends to Fig. 4

**Fig. 6:**
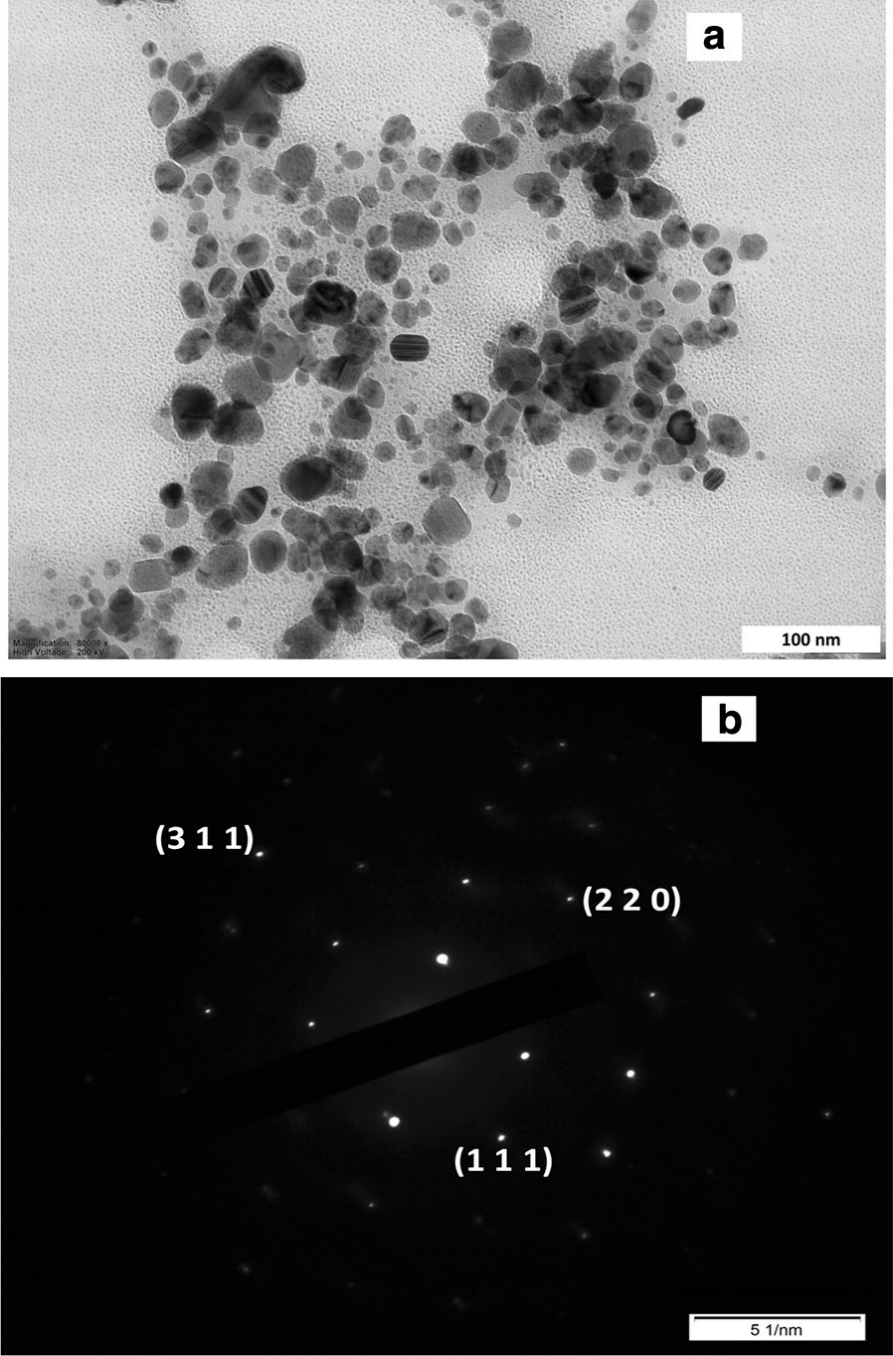
TEM images (**a**), SAED pattern (**b**) of Ag NPs obtained from *A. annua* extract. d-spacing values are 2.35, 1.45 and 1.23 Å for (1 1 1), (2 2 0) and (3 1 1) planes, respectively (**b**). For the others, see the legends to Fig. 2

The size, shape and morphological structure of NPs play an important role in their functionality and toxic effects on the environment and human organism (Prasad 2014; Griffin et al. 2018; Chokkareddy and Redhi 2018). Structural characteristics like size and shape of Ag NPs were investigated using TEM images, Nanosight and DLS analysis. The result of Nanosight analysis of biogenic synthesized Ag NPs confirmed that the reduced NPs were in the nano- sized range. The mode value of 3 separate measurements resulted in a main hydrodynamic diameter of 49.4 ± 3.9 nm and second peak with around 88 nm, demonstrating a distribution of particle sizes in this range (Fig. 4). This is in agreement with DLS measurements of the same sample that resulted in a hydrodynamic radius of 46.16 ± 8.56 nm (Fig. 5a). In addition, the Nanosight measurements revealed an integrated Ag NP concentration of 7 × 10^11^ particles mL^−1^. TEM images confirmed that these NPs had spherical shape with corresponding size (Fig. 6a), while also a few particle aggregates where observed (Fig. 6b). The selected-area electron diffraction pattern (SAED) of an individual Ag NPs proved its crystalline nature. The interplanar d-spacing values are 2.35, 1.45 and 1.23 Å for (1 1 1), (2 2 0) and (3 1 1) planes respectively and match the reported values for Ag NPs (Jyoti et al. 2016). The toxic effect was dependent on the size and shape of NPs, uptake of metal ions and their translocation in plant cells (Kulkarni and Muddapur 2014; Ovais et al. 2016; Rao and Tang 2017; Chokkareddy and Redhi 2018; Roy et al. 2019). It is known that the spherical particles lower toxicity more than dendritic particles (Kelly et al. 2003; Chokkareddy and Redhi 2018; Roy et al. 2019).

### Evaluation of various concentrations of Ag NPs against different bacteria

**Fig. 7:**
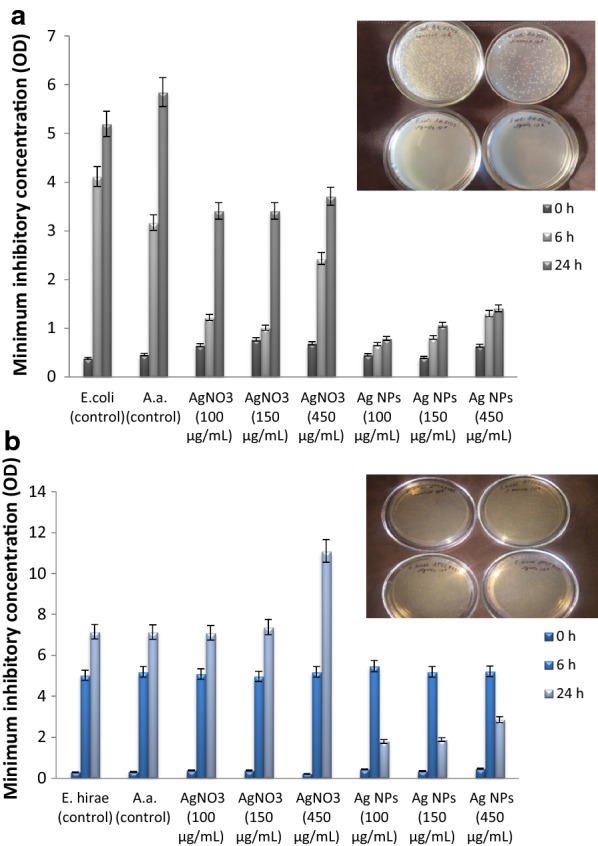
Antibacterial efficiency of Ag NPs tested on *E. coli* BW 25113 (**a**) and *E. hirae* ATCC 9790 (**b**) wild type strains. Ag NPs with concentration from 100 to 450 mg mL^−1^ were added into the bacterial growth medium. Data value was compared with the corresponding control value (p ≤ 0.05)

The antibacterial activity of the synthesized Ag NPs against the tested non-pathogenic bacteria was assessed on the growth of bacteria determining minimal inhibitory concentration (MIC) and on the cell viability (Poladyan et al. 2013). It has been observed that the Ag NPs demonstrated high antibacterial activity against Gram-negative and Gram-positive bacteria compared to the controls. As controls, bacteria + AgNO_3_ and bacteria + extract were used. The growth of both *E. coli* and *E. hirae* was inhibited at lower Ag NPs concentration (100 µg mL^−1^ and 150 µg mL^−1^, respectively) compared to the high concentration (450 µg mL^−1^). Our study showed that synthesized Ag NPs by *A. annua* extract had potential antibacterial activity, particularly they had appropriate effect on Gram-negative bacteria at low concentration (100 µg mL^−1^) (Fig. 7). This effect may be due to the bioactive metabolite sesquiterpene lactone artemisinin present in *Artemisia*. Several researchers (Gurunathan et al. 2014; Roy et al. 2019) reported that the NPs concentration directly correlates with bacterial species. They showed that the antibacterial activity of Ag NPs at low concentration is more effective against Gram-negative than against Gram-positive bacteria. In this regard, the cell viability was reduced and no growth at MIC values was observed for both strains. In case of *E. coli,* the synthesized Ag NPs not only inhibited bacterial growth, but also killed them after 24 h. However, the same concentration of Ag NPs led to 72% reduction of the Gram-positive bacterial population. The extract and AgNO_3_ (control) did not show high inhibitory effects on the cell viability. Although the *A. annua* and AgNO_3_ have antibacterial potential, however, their selected concentration did not show high antibacterial activity compared to the Ag NPs. Thus, the bactericidal effect depends on the concentrations, and it is specific for each bacterial strain.

The antibacterial activity of synthesized Ag NPs may be connected to the nano-sizes and spherical shape of the Ag NPs, NPs’ surface charge and also to the large surface area of Ag NPs, which makes them highly reactive and provides better interaction with bacterial cells (Sondi and Salopek-Sondi 2004; Vega-Jimenez et al. 2019). The positively charged Ag NPs show bactericidal and bacteriostatic activity (Roy et al. 2019). These parameters enable them to reach the nuclear content of bacteria easily. It is suggested that Ag NPs may attach to the surface of the bacterial cell membrane, disrupting membrane permeability and bacterial DNA replication (Sondi and Salopek-Sondi 2004; Bhabra et al. 2009; Chandran et al. 2014).

In case of both bacterial strains the growth of bacterial populations has decreased significantly at 100 µg mL^−1^ (*E. coli*), at 100 µg mL^−1^ and 150 µg mL^−1^ (*E. hirae*) on 6th and 24th h with respect to control (bacteria). Ag NPs at 450 µg mL^−1^ concentration also demonstrated antibacterial effect; however, the antibacterial activity of the Ag NPs at 100 µg mL^−1^ concentrations was slightly higher than at 450 µg mL^−1^ compared to the control; control was without NPs addition.

The number of bacterial colonies in various conditions were: *E. coli* (control)—850 × 10^8^ cell mL^−1^; *E. coli* & Ag NPs—0 × 10^8^ cell mL^−1^; *E. coli* & AgNO_3_—14 × 10^8^ cell mL^−1^; *E. coli* & extract—377 × 10^8^ cell mL^−1^ (Fig. 7a). *E. hirae* (control)—1320 × 10^8^ cell mL^−1^,; *E. hirae* & Ag NPs—370 × 10^8^ cell mL^−1^; *E. hirae* & AgNO_3_—465 × 10^8^ cell mL^−1^; *E. hirae* & extract—762 × 10^8^ cell mL^−1^ (Fig. 7b).

## Discussion

Antibacterial activity of NPs depends on their characteristics. Shape of NPs has a crucial role for exhibition of antibacterial activity. Recent studies revealed that the antimicrobial activity of spherical NPs is higher compared to the triangular ones (Chokkareddy and Redhi 2018; Qing et al. 2018; Roy et al. 2019), however the exact mechanism of how shape affects the antibacterial activity is still unclear. Some studies proved that the NPs with small size demonstrated high antibacterial activity (Chokkareddy and Redhi 2018; Roy et al. 2019). According to the literature (Prasad 2014; Roy et al. 2019) the antibacterial activity is increased with the increase of Ag NPs’ concentration and the time of their exposure to both Gram-negative and Gram-positive microorganisms. However, our findings demonstrated that an antibacterial activity against both bacterial strains is higher at low concentration of Ag NPs (see Fig. 7). The bars represent the NPs various concentrations effect on bacterial optical density (OD) changes during the growth (0, 6 and 24 h). Figure 7 shows the number of bacterial viable colonies grown in the presence of NPs as a function of initial bacterial suspension dilution (see Materials and methods). Several researchers explained the mechanism of Ag NPs’ impact on bacterial cells based on the intracellular physicochemical processes, particularly, the oxidation of protoplasm and its destruction by oxygen, while, the Ag NPs play the role of catalyst (Sondi and Salopek-Sondi 2004; Chandran et al. 2014). Effects on membrane permeability and membrane-associated key enzymes including the proton F_O_F_1_-ATPase can be main mechanisms of Ag NPs (Gabrielyan and Trchounian 2019). Formation of complexes of nucleic acid with heavy metals is due to impaired DNA stabilizing and bacterial viability (Sondi and Salopek-Sondi 2004; Vega-Jimenez et al. 2019). Ag does not affect the cells’ DNA directly; however, it increases the number of intracellular free radicals that reduce the concentration of intracellular active oxygen compounds (Sondi and Salopek-Sondi 2004; Bhabra et al. 2009; Wu et al. 2013). It was also assumed that interaction of Ag ions with ribosomes led to inhibition of enzymes and protein expression necessary for ATP production (Gurunathan et al. 2014; Bhabra et al. 2009; Vega-Jimenez et al. 2019). The mechanism of antibacterial effects of Ag NPs are still not clearly understood, but some studies have shown that Ag NPs may attach to the negatively charged bacterial cell wall and cleave it, which leads to protein denaturation and cell death (Sondi and Salopek-Sondi 2004; Wu et al. 2013; Chandran et al. 2014). Thus, probably the mechanism of Ag NPs action on bacterial cells is related to the fact that Ag NPs might be adsorbed on the cell membrane, therefore obstructing the growth of bacteria, and have protective function.

The results suggested that the biosynthesized Ag NPs using *A. annua* extract had spherical shape with diameter of 20–90 nm; they were formed at high pH values. Our findings showed that low concentration of biogenic Ag NPs led to increase in the antibacterial activity and could be used as an excellent source against tested bacteria; however, the bactericidal effect of biosynthesized NPs could be different depending on the organism tested. In spite of the high antibacterial activity of Ag NPs, we suggest that there is a need of further studies of their bactericidal effect on animal models before the use of synthesized NPs as antibacterial agents.

## Data Availability

All data analyzed during this study are included in this article. The data supporting the conclusions of this article are included within this article.

## Abbreviations

NPs: Nanoparticles
Ag NPs: Silver nanoparticles
SPR: Surface plasmon resonance
TEM: Transmission electron microscopy
DLS: Dynamic light scattering
SAED: Selected area electron diffraction
OD: Optical density
CFUs: Colony forming units
MIC: Minimal inhibitory concentration

## References

[CR1] Ansari Z, Saha A, Singha S, Sen K (2018). Phytomediated generation of Ag, CuO and Ag-Cu nanoparticles for dimethoate sensing. J Photochem Photobiol A Chem.

[CR2] Asif A, Muhammad K, Zaherr A, Hammad S (2015). Therapeutic potential of flavonoids and their mechanism of action against microbial and viral infections—a review. Food Res Int.

[CR3] Barabadi H, Mahjoub MA, Tajani B, Ahmadi A, Junejo Y, Saravanan M (2019). Emerging theranostic biogenic silver nanomaterials for breast cancer: a systematic review. J Cluster Sci.

[CR4] Bhabra G, Sood B, Fisher L, Cartwright L, Sanders M, Evans WH, Surprenant A, Castejon CL, Mann S, Davis SA, Hails LA, Ingham E, Verkade P, Lane J, Heesom K, Newson R, Case CP (2009). Nanoparticles can cause DNA damage across a cellular barrier. Nature Nanotechnol.

[CR5] Chandran K, Song S, Yun S (2014). Effect of size and shape controlled biogenic synthesis of gold nanoparticles and their mode of interactions against food borne bacterial pathogens. Arab J Chem.

[CR6] Chokkareddy R, Redhi GG (2018) Green synthesis of metal nanoparticles and its reaction mechanisms: synthesis, characterization and their applications. 113–139. 10.1002/9781119418900.ch4

[CR7] Davtyan GS (1980) Hydroponics. In: Reference book for chemicals used in agriculture. Kolos Publ. House, Moscow, pp. 382–385 **(in Russian)**.

[CR8] Gabrielyan L, Trchounian A (2019). Antibacterial activities of transient metals nanoparticles and membranous mechanisms of action. World J Microbiol Biotechnol.

[CR9] Gabrielyan L, Honvannisyan A, Gevorgyan V, Ananyan M, Trchounian A (2019). Antibacterial effects of iron oxide (Fe_3_O_4_) nanoparticles: distinguishing concentration-dependent effects with different bacterial cells growth and membrane-associated mechanisms. Appl Microbiol Biotechnol.

[CR10] Gouveia SC, Castilho PC (2013). *Artemisia annua* L: essential oil and acetone extract composition and antioxidant capacity. Ind Crops Prod.

[CR11] Griffin Sh, Masood MI, Nasim MJ, Sarfraz M, Ebokaiwe AP, Schafer K-H, Kack CM, Jacob C (2018). Natural nanoparticles: a particular matter inspired by nature. Antioxidants.

[CR12] Gurunathan S, Han JW, Kwon DN, Kim JH (2014). Enhanced antibacterial and anti-biofilm activities of silver nanoparticles against Gram-negative and Gram-positive bacteria. Nanoascale Res Lett.

[CR13] Iravani S, Zolfaghari B (2013). Green synthesis of silver nanoparticles using *Pinus eldarica* bark extract. BioMed Res Int.

[CR14] Jyoti K, Baunthiyal M, Singh A (2016). Characterization of silver nanoparticles synthesized using *Urtica dioica* Linn. leaves and their synergistic effects with antibiotics. J Radiat Res Appl Sci.

[CR15] Kelly KL, Coronado E, Zhao LL, Schatz GC (2003). The optical properties of metal nanoparticles: the influence of size, shape, and dielectric environment. J Phys Chem B.

[CR16] Kulkarni N, Muddapur U (2014). Biosynthesis of metal nanoparticles: a review. J Noanothechnol.

[CR17] Li Y, Guo Y, Yang Q, Weng XG, Yang L, Wang YG, Chen Y, Zhang D, Li Q, Liu XC, Kan XX, Chen X, Zhu XX, Kmoniekova E, Zidek Z (2015). Flavonoids casticin and chrysospenol D from *Artemisia anuua* L. inhibit inflammation in vitro and in vivo. Toxicol Appl Pharm.

[CR18] Nadeem M, Abbasi BH, Younas M, Ahmad W, Khan T (2017). A review of the green synthesis and anti-microbial applications of gold nanoparticles. Green Chem Lett Rev.

[CR19] Njagi EC, Huang H, Stafford L, Genuino H, Galindo HM, Collins JB, Hoag GE, Suib SL (2011). Biosynthesis of iron and silver nanoparticles at room temperature using aqueous *Sorghum bran* extracts. Langmuir.

[CR20] Ovais M, Khalil AT, Raza A, Khan MA, Ahmad I, Islam NU, Saravanan M, Ubaid MF, Ali M, Shinwari ZH (2016). Green synthesis of silver nanoparticles via plant extracts: beginning a new area in cancer theranostics. Nanomedicine.

[CR21] Poladyan A, Ayvazyan A, Vassilian A, Trchounian A (2013). Oxidative and reductive routes of glycerol and glucose fermentation by *Escherichia coli* batch cultures and their regulation by oxidizing and reducing reagents at different pH. Curr Microbiol.

[CR22] Polyakova NY, Polyakov AY, Sukhorukova IV, Shtansky DV, Grigorieva AV (2017). The defining role of pH in the green synthesis of plasmonic gold nanoparticles using *Citrus limon* extract. Gold Bull.

[CR23] Prasad R (2014). Synthesis of silver nanoparticles in photosynthetic plants. J Nanoparticles.

[CR24] Qing Y, Cheng L, Li R, Liu G, Zhang Y, Tang X, Wang J, Liu H, Qui Y (2018). Potential antibacterial mechanism of silver nanoparticles and the optimization of orthopedic implants by advanced modification technologies. Int J Nanomed.

[CR25] Rahman A, Kumar Sh, Bafana A, Dahoumane SA, Jeffryes C (2019). Biosynthetic conversion of Ag^+^ to highly stable Ag^0^ nanoparticles by wild type and cell wall deficient strains of *Chlamydomonas reinhardtii*. Molecules.

[CR26] Ram Sh (2011). Research output on *Artemisia* (*Artemisia annua*): a bibliometric study. Ann Lib Inform Stud.

[CR27] Rao B, Tang R (2017). Green synthesis of silver nanoparticles with antibacterial activities using aqueous *Eriobotrya japonica* leaf extract. Adv Nat Sci Nanosci Nanotechnol.

[CR28] Rastogi A, Zivcak M, Sytar O, Kalaji HM, He X, Mbarki S, Brestic M (2017). Impact of metal and metal oxide nanoparticles on plant. Crit Rev Front Chem.

[CR29] Raveendran P, Fu J, Wallen SL (2003). Completely “green” synthesis and stabilization of metal nanoparticles. J Am Chem Soc.

[CR30] Roy A, Bulut O, Some S, Mndal AK, Yilmaz MD (2019). Green synthesis of silver nanoparticle: biomolecule-nanoparticle organizations targeting antimicrobial activity. RSC Adv.

[CR31] Sahu N, Soni D, Chandraskekhar B, Satpute DB, Saravanadevi S, Sarangi BK, Pandey RA (2016). Synthesis of silver nanoparticles using flavonoids: hesperidin, naringin and diosmin, and their antibacterial effects and cytotoxicity. Int Nano Lett.

[CR32] Schramek N, Wang H, Romisch-Margl W, Keil B, Radykewicz T, Winzenborlein B, Beerhues L, Bacher A, Rohdich F, Gershenzon J, Liu B, Eisenreich W (2010). Artemisinin biosynthesis in growing plants of *Artemisia annua.* A ^13^CO_2_ study. Phytochemistry.

[CR33] Seil JT, Webster TJ (2012). Antimicrobial application of nanotechnology: methods and literature. Int J Nanomed.

[CR34] Silva N, Radhouani H, Goncales A, Arajo C, Rodriguez G, Igrejas G, Poeta P (2010). In vitro activity of ceftobiprole against Gram-positive and Gram-negative bacteria isolated from humans and animals. J Antimicrob Chemother.

[CR35] Singh AK, Srivastava ON (2015). One-step green synthesis of gold nanoparticles using black cardamom and effect of pH on its synthesis. Nanoscale Res Lett.

[CR36] Sondi I, Salopek-Sondi B (2004). Silver nanoparticles as antimicrobial agent: a case study of *E. coli* as a model for gram-negative bacteria. J Colloid Interface Sci.

[CR37] Song YJK, Kim BS (2009). Rapid biological synthesis of silver nanoparticles using plant extracts. Bioprocess Biophys Eng.

[CR38] Tarahovsky YS, Kim YA, Yagolnik EA, Muzafarov EN (2014). Flavonoid-membrane interactions: involvement of flavonoid-metal complexes in raft signaling. Biochim Biophys Acta.

[CR39] Vega-Jimenez AL, Vazquez-Olmos AR, Acosta-Gio E, Alvarez-Perez MA (2019) In vitro antibacterial activity evaluation of metal oxide nanoparticles. 10.5772/intechopen.84369

[CR40] Waclawek S, Goncukova Z, Adach K, Fijalkowski M, Cernik M (2018). Green synthesis of gold nanoparticles using *Artemisia dracunculus* extract: control of the shape and size by varying synthesis conditions. Environ Sci Pollut Res Int.

[CR41] Wu T, He M, Zang X, Zhou Y, Qui T, Pan S, Xu X (2013). A structure-activity relationship study of flavonoids as inhibitors of *E. coli* by membrane interaction effect. Biochim Biophys Acta.

